# Nanoencapsulation of Anthocyanins from Red Cabbage (*Brassica oleracea* L. var. *Capitata f. rubra*) through Coacervation of Whey Protein Isolate and Apple High Methoxyl Pectin

**DOI:** 10.3390/antiox12091757

**Published:** 2023-09-13

**Authors:** Ilaria Fierri, Laura De Marchi, Roberto Chignola, Giacomo Rossin, Maria Bellumori, Anna Perbellini, Ines Mancini, Alessandro Romeo, Gloria Ischia, Asia Saorin, Federica Mainente, Gianni Zoccatelli

**Affiliations:** 1 Department of Biotechnology, University of Verona, Strada Le Grazie 15, 37134 Verona, Italy; ilaria.fierri@univr.it (I.F.); laura.demarchi_01@univr.it (L.D.M.); roberto.chignola@univr.it (R.C.); giacomo.rossin@univr.it (G.R.); anna.perbellini_04@studenti.univr.it (A.P.); asia.saorin@univr.it (A.S.); federica.mainente@univr.it (F.M.); 2 Department of NEUROFARBA, University of Florence, Via Ugo Schiff 6, Sesto F.no, 50019 Florence, Italy; maria.bellumori@unifi.it; 3 Department of Physics, University of Trento, Via Sommarive 14, Povo, 38123 Trento, Italy; ines.mancini@unitn.it; 4 Department of Computer Science, University of Verona, Strada Le Grazie 15, 37134 Verona, Italy; alessandro.romeo@univr.it; 5 Department of Industrial Engineering, University of Trento, Via Sommarive 9, Povo, 38123 Trento, Italy; gloria.ischia@unitn.it

**Keywords:** red cabbage, encapsulation, acylation, anthocyanins, whey protein isolate, high-methoxyl pectin, coacervation, nanoparticles, antioxidant, by-products

## Abstract

Encapsulation is a valuable strategy to protect and deliver anthocyanins (ACNs), phenolic compounds with outstanding antioxidant capacity but limited stability. In this study, coacervation was used to encapsulate an ACN-rich red cabbage extract (RCE). Two agri-food by-product polymers, whey protein isolate (WPI) and apple high-methoxyl pectin (HMP), were blended at pH 4.0 in a specific ratio to induce the formation of nanoparticles (NPs). The process optimisation yielded a monodispersed population (PDI < 0.200) of negatively charged (−17 mV) NPs with an average diameter of 380 nm. RCE concentration influenced size, charge, and antioxidant capacity in a dose-dependent manner. NPs were also sensitive to pH increases from 4 to 7, showing a progressive breakdown. The encapsulation efficiency was 30%, with the retention of ACNs within the polymeric matrix being influenced by their chemical structure: diacylated and/or C3-triglucoside forms were more efficiently encapsulated than monoacylated C3-diglucosides. In conclusion, we report a promising, simple, and sustainable method to produce monodispersed NPs for ACN encapsulation and delivery. Evidence of differential binding of ACNs to NPs, dependent on specific acylation/glycosylation patterns, indicates that care must be taken in the choice of the appropriate NP formulation for the encapsulation of phenolic compounds.

## 1. Introduction

Anthocyanins (ACNs) are plant secondary metabolites belonging to the flavonoids family, which represents the most numerous group of phenolic compounds [1,2]. Their structure, which is based on the flavylium cation (2-phenyl-1-benzopyrilium), presents several hydroxyl (-OH) and/or methoxyl (-OCH_3_) groups linked to one or more sugars. The number and position of the different substituents give rise to the six basic anthocyanidins: cyanidin, malvidin, pelargonidin, petunidin, delphinidin, and peonidin, which, in turn, may additionally present acylations on the hydroxyl group of their sugar units. The most interesting feature of ACNs is that they are natural water-soluble red pigments responsible for the colour of several fruits and vegetables such as berries, red cabbage, black carrot, purple corn, and potatoes [2]. Thus, from a technological point of view, ACNs play a crucial role in guiding the acceptance of plant-derived foods by consumers and represent natural alternatives to synthetic food colourants [3]. The importance of ACNs goes well beyond their technological implications since, in the last few years, several health-promoting properties have been ascribed to these molecules. ACNs possess antioxidant and anti-inflammatory potency and have been proven effective in preventing age-related chronic diseases such as cardiovascular disease (CVD), cancer, neurodegenerative diseases, and eye-related diseases [4,5].

Nevertheless, the chemical stability of ACNs is scarce and influenced by several physiochemical parameters such as temperature, oxygen, light, the presence of metals, and degrading enzymes [6]. For this reason, processes typically applied to transform and conserve foods may strongly affect the colour and biological activity of ACNs. To tackle this problem, encapsulation strategies based on different wall materials to protect ACNs have been implemented [7,8,9]. In addition, being efficacious, an ideal encapsulation process should also be cost-effective and based on sustainable materials, possibly achieved from food by-products from a circular economy perspective [10]. Whey proteins and pectins, derived from cheese-making and fruit-juice production chains, respectively, represent good candidates to encapsulate phenolic compounds. Previously published studies showed that these two polymers can electrostatically interact in a specific pH range to form a network that can entrap polyphenols, among which are ACNs [11,12,13]. The interactions of these colloids with phenolic compounds are mainly driven by hydrogen bonds and electrostatic interactions. Depending on the different conditions, this process, also called coacervation, allows for the production of nanoparticles that can be used to protect and deliver ACNs [11]. The results reported so far are limited to applications on simple anthocyanins, such as cyanidin-3-glucoside, or are referred to as ACNs-rich extracts deriving from undisclosed plant sources. However, ACNs are known to present many structures that can strongly affect their functional properties. To verify whether the structure of the ACNs may influence the performance of the process in terms of efficiency, different ACNs should be tested under the same conditions. For this reason, red cabbage (RC) represents a suitable model since it contains several ACNs characterised by different acylations and glycosylations, which are also acknowledged for their positive biological activities [14]. The aim of the present study was to investigate the optimal conditions for the encapsulation of RC ACNs within a whey protein isolate (WPI) and high methoxyl pectin (HMP) coacervate. The effects of ACN concentration on the physiochemical characteristics of the beads and whether differently acylated and glycosylated ACNs might specifically interact with the polymeric network were evaluated.

## 2. Materials and Methods

### 2.1. Materials

Fresh red cabbage (*Brassica oleracea* L. var. *Capitata f. rubra*, cultivar Cairo) was purchased from a local market. Whey protein isolate (WPI) was purchased from Carbery (Cork, Ireland). High methoxyl pectin (70–75% DE) from apple skin (HMP), Folin-Ciocalteau reagent, sodium carbonate, 2,2′-azinobis-3-ethyl-benzothiazoline-6-sulfonic acid (ABTS), 2,4,6-tripyridyl-s-triazine (TPTZ), ferric chloride, formic acid, acetonitrile, methanol, hydrochloric acid, cyanidin 3-glucoside (Cy3G), gallic acid (GA), Trolox, and ethanol were purchased from Sigma-Aldrich (St. Louis, MO, USA).

### 2.2. Preparations and Characterisation of Anthocyanin Water Extract

#### 2.2.1. ACNs Extraction

The extraction of ACNs from RC was conducted following the protocol suggested by Zanoni et al. (2020) [15] with some adaptations. Briefly, 500 g of leaves were collected from daily-fresh samples harvested at maturity and without signs of pest or disease injury, minced with a knife, and homogenised using a mixer. The extraction was conducted using deionised water acidified with formic acid (final pH 3.4 ± 0.1) for 60 min at room temperature under steady agitation (ratio sample: water 1:1). RC extract (RCE) was finally centrifuged at 6000× *g* at 8 °C for 15 min using a Beckman Coulter Avanti JXN-26 supercentrifuge (Beckman Coulter Inc., Brea, CA, USA), and the supernatant was freeze-dried using a 5Pascal Lio5P (Milan, Italy) freeze-drying apparatus. The process was conducted at −50 °C for at least three days with a final chamber pressure of 0.020 mbar. The resulting samples were stored at −20 °C until further use.

#### 2.2.2. Differential pH Method

Free anthocyanin concentration was measured through the differential pH method as described by the AOAC Official Method 2005.02 [16]. In brief, RCE samples were diluted in potassium chloride buffer (pH 1.0) and sodium acetate buffer (pH 4.5) in a 1:20 ratio (*v*:*v*). The absorbance of each solution was then measured using a Jasco V-730 Spectrophotometer (Jasco Europe, Cremella, Italy) at 520 nm and 700 nm. The free anthocyanin content was calculated using the following equation:

(1) $$\mathrm{Free}\mathrm{anthocyanins}(\mathrm{mg}/L)=\frac{A\times \mathrm{MW}\times \mathrm{DF}\times 10^{3}}{\varepsilon \times l}$$

where A = (A520 nm–A700 nm) at pH 1.0—(A520 nm–A700 nm) at pH 4.5; MW (molecular mass) = 449.2 g/mol for cyanidin-3-glucoside (Cy3G); DF = dilution factor established; *Ɛ* (molar extinction coefficient) = 26,900 L mol^−1^ cm^−1^ for Cy3G. The analysis was performed in 1 cm path length cuvettes (*l*).

#### 2.2.3. HPLC-DAD-MS Analysis

Chromatographic analyses were carried out by an HPLC Extrema LC-4000 system (Jasco Europe Srl) equipped with a Zorbax Eclipse Plus C18 column (i.d. 4.6 × 250 mm, 5 μm particle size, Agilent Technologies, Palo Alto, CA, USA). The mobile phases were as follows: (A) 5% formic acid and (B) acetonitrile, and the analyses were carried out by applying the following multistep gradient at 0.5 mL/min: 5–21% B (0–76 min), 21–100% B (76–78 min), 0–100% B (78–80 min), 100–4% B (82–84 min). A six-point Cy3G calibration curve (10–200 ng) was prepared. Peak areas were linearly correlated with corresponding concentrations (R^2^ = 0.999, *p* << 0.001). Data acquisition was performed using Jasco ChromNAV v.2.03.06 software.

ACNs identification was performed using an HP 1260 L liquid chromatography equipped with a DAD detector (Agilent Technologies, Palo Alto, CA, USA) and an API (Atmospheric Pressure Ionisation) interface/ESI (ElectroSpray Ionization) using the same column and conditions described for HPLC-DAD analysis. MS operating conditions were as follows: N_2_ flow of 10.5 L/min; gas temperature of 350 °C; capillary voltage of 3500 V; nebuliser pressure of 1811 Torr. The acquisition of the spectra was performed in positive ionisation, with a fragmentation energy of 150 eV.

### 2.3. Nanoparticles Formation

#### 2.3.1. Solutions Preparation

WPI and pectin solutions were prepared by adapting the protocol proposed by Thongkaew and co-workers [12]. Briefly, powdered WPI was solubilised at 5% (*w*/*v*) concentration, while HMP was solubilised at 2% (*w*/*v*) with deionised water and stirred at ambient temperature to ensure complete hydration. WPI solution was filtered using filter paper of 5–8 μm size (Lab Logistic Group, Meckenheim, Germany) to remove undissolved particles. All solutions were stored at 4 °C until use and for no more than 2 days.

#### 2.3.2. WPI/HMP Complex Coacervation

Nanoparticles were obtained by combining WPI and HMP as described previously with some modifications [11]. Four blending methods were tested and compared (see Table 1). For methods Nos. 2 and 3, the WPI solution was heated at 65 °C for 40 min, as previously described [12], while for method No. 4, the heating process involved the whole mix. No heat treatments were performed for Method No. 1. Attempts to heat the WPI solution at 90° C for 5 min, as described by Arroyo-Maya et al. (2015) [11], led to its coagulation as a result of protein denaturation and aggregation. In all cases, after the preparation, the pH was brought to 4.0 with a 1 N HCl solution to allow the coacervation. All formulations were centrifuged using an Eppendorf 5424 R centrifuge (Hamburg, Germany) at 21,000× *g* for 90 s at room temperature to remove macroaggregates. Control samples were prepared following the same procedures, substituting RCE with an equal volume of deionised water.

### 2.4. Dynamic Light Scattering (DLS) Analysis

DLS analyses were performed using a Malvern Zetasizer Nano-ZS (Malvern Instruments, Worcestershire, UK) at 25° C. Small aliquots of WPI, HMP, and fresh NP formulations were opportunely diluted with 10 mM sodium acetate buffer at pH 4.0 to avoid multiple scattering effects. Each DLS analysis was performed in triplicate using automatic settings and disposable cuvettes. The DLS outputs considered in this study were the Z-average, which represents an intensity-weighted mean of all the particle populations; the hydrodynamic diameter of the main peak; the polydispersity index (PDI), which indicates the variance in the size of the particle distribution; and the ζ-potential, which defines the particle charge. The latter parameter was measured using the Smoluchowsky approximation.

### 2.5. Encapsulation Efficiency (EE)

The encapsulation efficiency was measured through HPLC-DAD analysis of the loaded ACNs after the removal of unbound ACNs and precipitation of the polymeric material. Briefly, 1.5 mL of fresh formulation (at 1042.0 mg/L) was dialysed for 24 h using an MD10 Membra-Cel^®^ Membrane Dialysis to separate unbound ACNs from the biopolymer particles. Dialysis was performed at 4 °C against 1 L of 5 mM sodium acetate buffer adjusted to pH 4.0 under gentle stirring. An aliquot of dialysed sample was treated with EtOH at a ratio of 1:1 *v*/*v*, gently agitated on an orbital shaker (Heidolph, Germany) for 10 min to cause the denaturation of the protein nanoparticles, and ultimately centrifuged to remove the insolubilised polymers. The supernatant was dried in a Heto Vacuum Centrifuge (Thermo-Fisher, Waltham, MA, USA), resuspended in 5% formic acid, and quantified by HPLC. This ACNs fraction represents “bound ACNs," while “total ACNs” are represented by the undialysed sample treated the same way. Hence, the EE% can be calculated using the following equation:

(2) $$\mathrm{EE}(\%)=\frac{\mathrm{bound}\mathrm{ACNs}}{\mathrm{total}\mathrm{ACNs}}\times 100$$

Each analysis was performed in triplicate.

### 2.6. Effect of pH

The stability of the NPs was tested by DLS after diluting each sample in 40 mM acetate buffer at pH 5.0- or 40-mM phosphate buffer at pH 6.0 and 7.0 and gently shaking for 10 min. Samples were further centrifuged and analysed as described in Section 2.4.

### 2.7. Antioxidant Capacity

#### 2.7.1. ABTS

The ABTS radical-scavenging assay was performed following the method proposed by Bellumori et al. (2021) [17] with some adaptation. The ABTS working solution was produced by mixing a 7 mM ABTS solution with a 2.6 mM potassium persulfate solution in equal quantities and allowing the mixture to react for 12 h protected from light. The radical solution was then diluted with 10 mM sodium acetate buffer, pH 4.0, to an absorbance of 0.75 ± 0.02 at 734 nm immediately before the analysis. The assay was performed on 96-well microplates (Starstedt, Germany), mixing 20 μL of standard extract or NPs with 200 μL of ABTS and subsequently incubating in the dark for 10 min. The absorbance decrease at 734 nm was measured through a microplate reader. The results were expressed as Trolox-equivalent antioxidant capacity (TEAC, in mM) using a Trolox calibration curve from 0.5 mM to 0.015 mM.

#### 2.7.2. Ferric-Reducing Antioxidant Power (FRAP)

The FRAP assay was conducted on a 96-well microtiter plate, adapting a previously proposed protocol [18]. Shortly, 20 μL of standard, extract, or NPs were mixed with 280 μL of freshly prepared FRAP reagent. The plates were incubated at 37 °C for 30 min, and the absorbance was read at 593 nm. The FRAP reagent was prepared by mixing 10 volumes of 300 mM acetate buffer at pH 4.0, 1 volume of 2,4,6-tripyridyl-s-triazine (TPTZ) at 10 mM, and 1 volume of 20 mM ferric chloride solution in 40 mM HCl. The results were expressed as Trolox equivalents (mM) using a Trolox calibration curve from 1 mM to 0.015 mM.

### 2.8. Fourier Transform Infrared Spectroscopy (FTIR)

The IR spectra of WPI, HMP, RCE, and NPs were obtained using the Jasco FT/IR-4700 type A (S/N F058261788 version 2.23.06) FTIR spectrometer equipped with a horizontal attenuated total reflectance (ATR) crystal (Diamond). The spectra were collected in transmittance mode on lyophilised or dried samples placed directly onto the ATR crystal. Each spectrum is the result of the average of 64 scans at 4 cm^−1^ resolution. Measurements were recorded between 4000 and 400 cm^−1^. Images were recorded and analysed using the dedicated software, Jasco FTIR-4700 Spec. Magr 2020.

### 2.9. Atomic Force Microscopy (AFM)

The AFM analysis was performed on an NT-MDT Solver Pro AFM (Moscow, Russia) in semi-contact mode, using a rectangular cantilever equipped with an NT-MDT NSG01 crystal silicon golden coated tip. NP solutions were diluted 1:30 in 10 mM sodium acetate buffer at pH 4.0 immediately before analysis. A 50 μL drop of suspension was deposited onto freshly cleaved inert mica supports and incubated for 10 min at ambient temperature, then dried under argon gas flow. Images were processed with the open-source software Gwyddion ver. 2.62.

### 2.10. Transmission Electron Microscopy (TEM)

The analysis was conducted using an analytical field emission gun scanning transmission electron microscope (FEG-STEM) Thermo Fisher Scientific Talos F200S (Thermo Fisher Scientific, Waltham, MA, USA) at a voltage of 100 KeV. NP solutions were diluted 1:50 in 10 mM sodium acetate buffer at pH 4.0 and deposited onto carbon-coated Formvar grids. Images of the particles were collected in S/TEM mode using the BrightField (BF) and the High Angle Annular Dark-Field (HAADF) detectors.

### 2.11. X-ray Powder Diffraction (XRPD)

XRPD data collection was carried out on an Italstructures IPD3000 instrument (GNR Analytical Instruments Group, Novara, Italy), equipped with a Cu-anode source (40 kV, 30 mA) coupled to multilayer parallel beam optics to suppress k-beta radiation and a 1024-channel Dectris Mythen 1 K hybrid pixel detector. Powder patterns were acquired in the 13°–80° 2θ range with a 0.02° step for a total of 2400 s of acquisition time per sample. Powder samples were loaded in a ring-shaped hollow sample holder, compacted by the back-loading technique, and finally placed in reflection geometry on a spinning support to optimise sampling statistics.

### 2.12. Statistical Analysis

Statistical analyses were performed using GraphPad Prism version 8.0.0 (GraphPad Software, San Diego, CA, USA), and the data were expressed as mean ± standard error of the mean (SE). Differences between the experimental groups were assessed using the Student’s *t*-test, the unequal variances *t*-test (Welch test), and, where appropriate, the one-way analysis of variance (ANOVA), followed by the post-hoc Tukey test.

## 3. Results and Discussion

### 3.1. Characterisation of RCE

**Figure 1:**
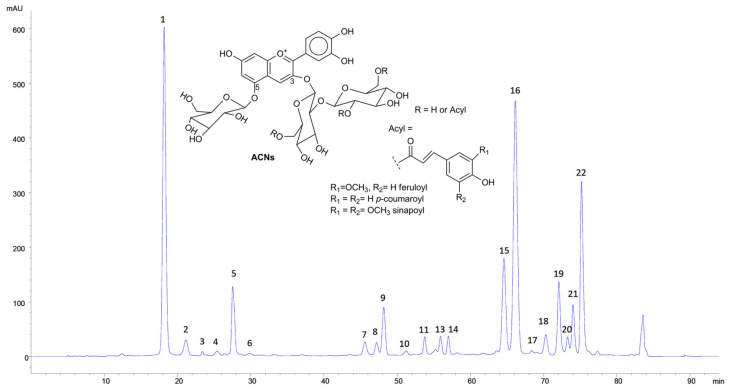
HPLC-DAD chromatogram of RCE (520 nm). Peaks identification is as reported in Table 1. Inset: typical structure of RC ACNs.

The concentration of free ACNs was 577.9 ± 70.5 mg/L. This value is consistent with previously published results [14]. RCE was analysed by HPLC-DAD, which allowed for the detection of 22 peaks compatible with ACNs (Figure 1). The analysis by HPLC-MS led to the identification of 16 peaks as glycosylated and differently acylated ACNs, whose tentative identification is displayed in Table 1. The identification was carried out by comparing the data with the analytical standards when available, their retention times, and their mass spectral characteristics with the literature [19,20,21,22].

The chromatogram is similar to previously published results [20], even if the relative abundance of the different ACNs showed some differences. This was not surprising given the variability of the plant material in terms of genotypes, season, cultural practices, etc. [23].

**Table 1:** Identification of RC ACNs.

Peak No.	Peak Identity	M^+^ (*m*/*z*)
1	Cyanidin-3-diglucoside-5-glucoside	773
2	Cyanidin-3-glucoside-5-glucoside	611
5	Cyanidin-3-(sinapoyl)-diglucoside-5-glucoside	979
5	Cyanidin-3-(sinapoyl)-triglucoside-5-glucoside	1141
7	Cyanidin-3-(p-coumaroyl)-triglucoside-5-glucoside	1081
8	Cyanidin-3-(feruloyl)-triglucoside-5-glucoside	1111
9	Cyanidin-3-(sinapoyl)-triglucoside-5-glucoside	1141
11	Cyanidin-3-(feruloyl)(feruloyl)-triglucoside-5-glucoside	1287
13	Cyanidin-3-(feruloyl)(sinapoyl)-triglucoside-5-glucoside	1317
14	Cyanidin-3-(sinapoyl)(sinapoyl)-triglucoside-5-glucoside	1347
15	Cyanidin-3-(p-coumaroyl)-diglucoside-5-glucoside	919
16	Cyanidin-3-(sinapoyl)-diglucoside-5-glucoside	979
17	Cyanidin-3-(feruloyl)-diglucoside-5-glucoside	949
18	Cyanidin-3-(sinapoyl)-glucoside-5-glucoside	817
19	Cyanidin-3-(feruloyl)(feruloyl)-diglucoside-5-glucoside	1125
20	Cyanidin 3-(5-OHferuloyl)(sinapoyl)-triglucoside-5-glucoside	1171
21	Cyanidin-3-(feruloyl)(sinapoyl)-diglucoside-5-glucoside	1155
22	Cyanidin-3-(sinapoyl)(sinapoyl)-diglucoside-5-glucoside	1185

### 3.2. Coacervation

#### 3.2.1. Electrical Characterisation of the Biopolymers

**Figure 2:**
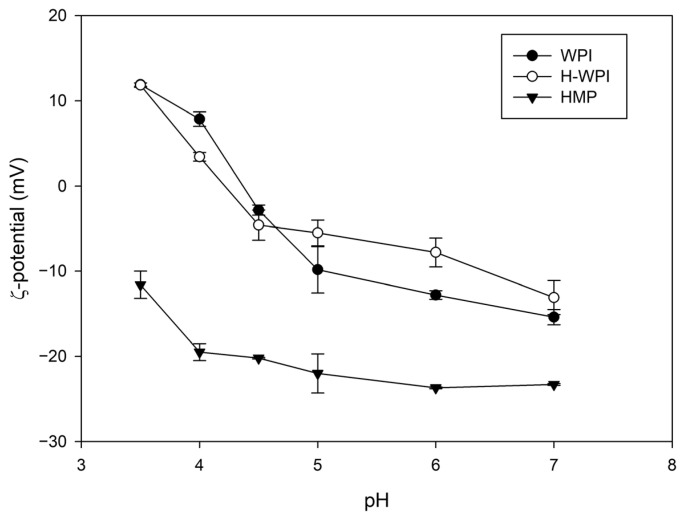
Influence of pH on the ζ-potential of WPI, pre-heated WPI (H-WPI), and HMP solutions.

The ζ-potential of the WPI solution was recorded in a pH interval ranging from 3.5 to 7.0. The isoelectric point of the protein mixture was around 4.4, in agreement with previously published data [24]. Heating WPI solutions above denaturation temperature has been demonstrated to improve the stability of electrostatically-stabilised complexes with pectin [25,26]. In our hands, heating at 90 °C, as previously described [11,25], led to a strong protein denaturation, which culminated in the coagulation of the WPI solution making further analyses impractical. The different behaviour observed by the authors has probably been caused by the 10-fold lower protein concentration they employed. On the contrary, the thermal treatment performed at a lower temperature (65 °C), as proposed by Thongkaew et al. (2014) [12], induced a steep decrease of the ζ-potential around pH 4.0 compared to the non-heated sample (Figure 2); however, no coagulation was observed.

The ζ-potential of HMP was also investigated. As expected, the polysaccharide displayed a negative charge independently of the pH, showing an increase below pH 4.0 due to the partial protonation of carboxyl groups of galacturonic acid, the main constituent of the pectin backbone. For this reason, the coacervation was performed at pH 4.0 since this specific value guaranteed the largest potential difference between the two colloids.

#### 3.2.2. Coacervation

Coacervation is an encapsulation method in which one or more polyelectrolytes bind an active ingredient, spontaneously separating within the formulation [27]. In this work, the coacervation process involved a protein solution, mainly composed of β-lactoglobulin (~56%) and, to a lesser extent, α-lactalbumin, and an anionic polysaccharide, i.e., apple pectin, which is characterised by nearly 70% methyl esterification. As suggested by Thongkaew et al. (2014) [12], the WPI:HMP ratio was 5:2, with a concentration of the biopolymer being 5–10 times higher than what can be found in the literature [11,28,29]. The increase in the polymer content was justified by the need for a more sustainable formulation. Indeed, too-diluted preparations need longer and more expensive concentration steps that might limit future scale-up. Higher polymer concentrations were impractical due to the excessive viscosity of the colloidal dispersion. Different heating and blending orders of the components were tested, and the stability of each formulation was assessed through the analysis of particle size, polydispersity index, and ζ-potential. The results are presented in Table 2.

**Figure 3:**
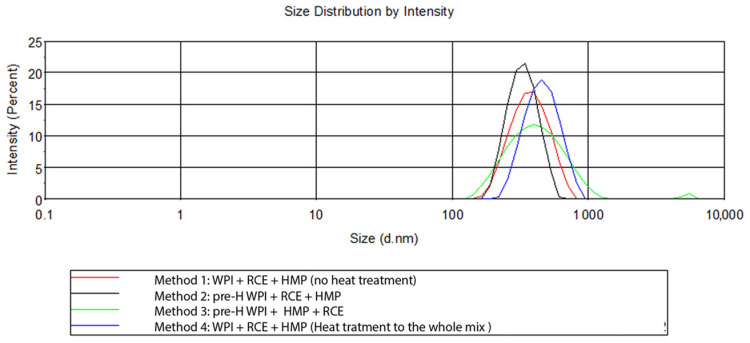
Representation of the DLS intensity % distribution of the formulations obtained with the four methods. Different lowercase letters indicate significant differences (*p* ≤ 0.05).

No significant differences in terms of ζ-potential between the methods were observed (*p* < 0.05). Although these values are somewhat similar to previously published data [11], the PDI of method No. 2 (0.170) is worthy of note since values below 0.200 are typical of a monodisperse particle population. Indeed, PDI is a measure of the heterogeneity of a sample based on size and varies from 0 to 1. The homogeneity of an NP formulation is considered an important quality trait as functional properties and stability depend on the dimension of the particles; therefore, the lower the dispersion, the better the functionality of the formulation. In this regard, method No. 3, which differs from the previous ones only for the blending order, i.e., the addition of HMP before RCE, showed the highest PDI. This is also evident by the presence of a second peak in the micrometre range displayed in Figure 3 (green line), in which examples of the size distributions of the different methods are presented. The same figure shows that NPs produced by method No. 4 were characterised by the biggest mean diameter (445 nm vs. 370–380 nm). This is in agreement with the results of Arroyo-Maya et al. (2015) [11], who observed an increase in the NP mean diameter when heating was carried out after the addition of the anthocyanin-rich extract. The absence of any thermal treatment (method No. 1) returned a relatively homogeneous NP population around 380 nm in size with a PDI slightly higher than 0.200 but with several precipitates or sediments. The diameter of the NPs is generally higher than those previously reported [11]; however, this might depend on the concentration of the polymers, which was higher than that used in previous works. Method No. 2 was thus selected for the following experiments.

#### 3.2.3. Effect of RCE Concentration on the Morphology of NPs

**Figure 4:**
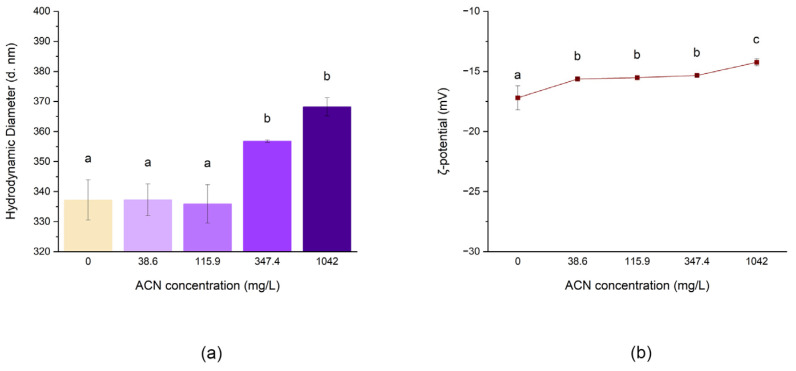
Effect of RCE concentration on the hydrodynamic diameter of the main peak (**a**) and ζ-potential (**b**) of the preparation obtained with method No. 2. Different lowercase letters indicate significant differences (*p* ≤ 0.05).

Lyophilised RCE was reconstituted in deionised water immediately before the preparation of each formulation. Different concentrations, namely 38.6, 115.9, 347.4, and 1042.0 mg/L, were used to formulate NPs following method No. 2. The empty coacervate was prepared using an equal amount of water. The results are shown in Figure 4 and Table 3.

The results show that the addition of RCE to the biopolymers caused an increase in the NP size. In particular, samples prepared at 347.4 mg/L and 1042.0 mg/L ACNs concentrations display a significantly larger hydrodynamic diameter than the less concentrated formulations (*p* < 0.05), whose size is close to the empty counterpart. Moreover, the ζ-potential was influenced by the presence of RCE in a dose-dependent manner (Figure 4), with NP negative charge decreasing significantly following the increase in RCE concentration. These results might be explained by considering that, at pH 4.0, the charge of ACNs can still present a faint cationic nature [30]. Under this hypothesis, the ACN charge could be neutralised by the negative charge of HMP, thus reducing the interaction of this colloid with WPI. This would decrease the overall coacervate final charge and lead to a looser polymeric network, thus increasing the dimension of the NPs. These values are similar to what Arroyo-Maya and McClements [11] reported, despite being less negative, likely due to the different pectin used. Generally, ζ-potentials lower than −30 mV or greater than +30 mV designate stable conditions to avoid nanoparticle aggregation, thus leading to more stable formulations. The low ζ-potential recorded in this case suggests its tendency to flocculation or aggregation while in liquid form and could thus benefit from a dehydration process that would turn the colloidal suspension into powder.

**Figure 5:**
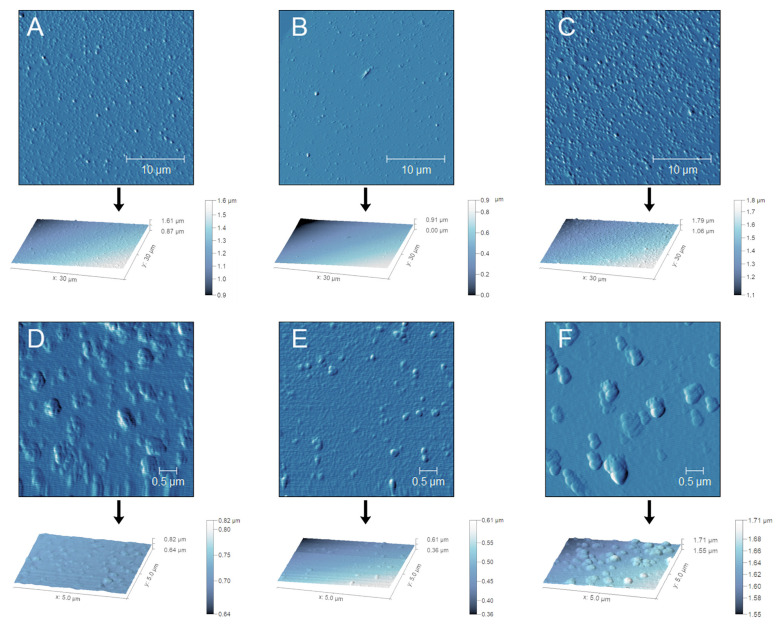
Semi-contact AFM analysis of empty NPs (**A**,**D**), NPs containing 38.6 mg/L ACNs (**B**,**E**), and NPs containing 1042.0 mg/L ACNs (**C**,**F**) The dimensions of the scanning areas were 30 × 30 for panels (**A**–**C**) and 5 × 5 μm for panels (**D**–**F**). Samples were diluted 1:30 using 10 mM sodium acetate buffer at pH 4.0 and allowed to be set on muscovite micas for at least 10 min before drying through argon flow immediately before scanning.

**Figure 6:**
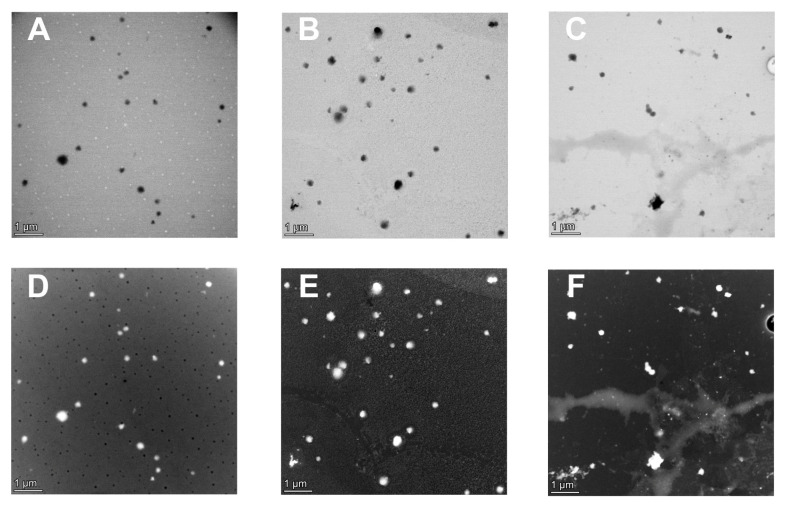
S/TEM images of empty NPs (**A**,**D**), NPs containing 38.6 mg/L ACNs (**B**,**E**), and NPs containing 1042.0 mg/L ACNs (**C**,**F**). Image samples were diluted 1:50 using 10 mM sodium acetate buffer at pH 4.0 and allowed to be set on carbon-coated Formvar grids at ambient temperature.

The results obtained by AFM and TEM are in agreement with the DLS analysis (Figure 5 and Figure 6). Although NPs showed a monodispersed distribution in liquid form, as described in Table 3, they tend to aggregate once the solvent is removed. With respect to AFM results, the dried samples showed a slight increase in size on the x and y axes and appeared flattened on the z axis (Figure 5). The different dimensions recorded in this instance may result from the loss of spherical shape suffered by the samples once dried, likely due to the NP’s soft structure.

#### 3.2.4. Effect of pH

**Figure 7:**
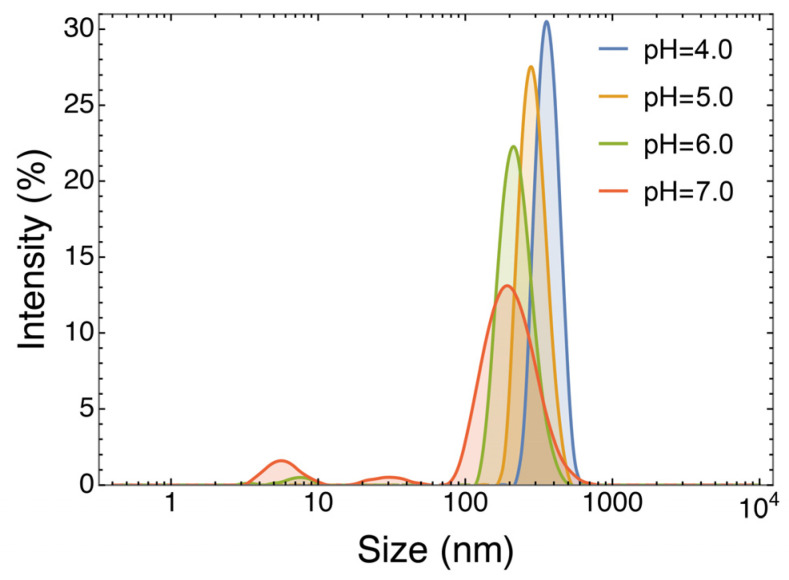
Overlayed DLS% intensity signals of particle distributions of the sample formulated at 1042.0 mg/L ACNs at pH 4.0, 5.0, 6.0, and 7.0.

Coacervation heavily relies on the electrostatic forces between oppositely charged biopolymers. Thus, pH changes can trigger the disruption of the polymeric network and the release of the trapped target molecules [31]. To evaluate this possibility, the NPs at 1042.0 mg/L ACNs were incubated at increasing pH values, and the effects were assessed by DLS analysis (Figure 7). At pH 4.0, which represents the pH value of the coacervation at its optimal conditions, results show a monodisperse NP population (PDI: 0.170, from Table 2). At pH 5.0, the PDI starts rising (PDI > 0.700), showing signs of coacervate disruption and an increase in the variance of particle distributions. This can also be deduced from the decrease in the signal intensity and the main peak diameter. The same trend can be observed at pH 6.0 (PDI > 0.300), where a substantial decrement in the signal intensity can be appreciated, which becomes even more evident at pH 7.0 (PDI > 0.500).

These results demonstrate that by increasing pH, the coacervates disaggregate. This phenomenon is of great importance since, together with the action of digestive enzymes, it might contribute to the release of the RCE active molecules within the gut lumen, where the pH is around 7.0 [32], improving their bioaccessibility.

#### 3.2.5. Antioxidant Capacity

**Figure 8:**
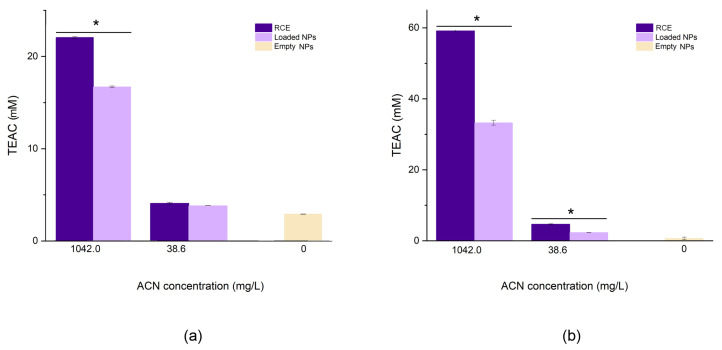
Comparison of the antioxidant capacity measured by ABTS (**a**) and FRAP (**b**) of NPs (at 38.6 mg/L and 1042.0 mg/L ACNs), RCE (at the same ACNs concentration), and empty NPs The * denotes significant differences (*p* < 0.05) between extract and coacervates at the same ACNs concentration.

Thanks to their peculiar structure, ACNs can exploit their antioxidant capacity to scavenge radicals following both hydrogen atom transfer (HAT) and single-electron transfer (SET) mechanisms [33]. Due to the complexity of these phenomena, their dependence on the environment, and their simultaneous occurrence, several bioassays are used to determine the total antioxidant capacity of polyphenols. In this study, two well-known assays based on different scavenging mechanisms have been employed, i.e., ABTS and FRAP, representative of HAT and SET mechanisms, respectively. The ABTS assay is based on the decolourisation (inhibition) of a blue/green radical chromophore mediated by the antioxidant. The inhibiting capacity of RCE was compared with that of empty and loaded NPs. The loaded coacervates at 1042.0 mg/L showed a lower TEAC (16.07 ± 0.1 mM) than the extract tested at the same concentration (TEAC: 22.05 ± 0.01 mM), probably due to the interaction between the biopolymers and the ACNs that limited the scavenging capacity of the latter (Figure 8a). Moreover, empty NPs displayed a noticeable AOC (2.91 ± 0.1 mM), which is similar to the AOC exerted by the sample containing 38.6 mg/L of ACNs. This is not surprising since it is acknowledged that proteins possess antioxidant capacity and may react with ABTS^•+^ as already observed [34]. This phenomenon is probably the reason why, at 38.6 mg/mL, non-significant differences were noted between coacervate and RCE. A similar scenario could be observed for the FRAP assay (Figure 8b). This is based on the reduction of the ferric ion mediated by electron-donating groups of the antioxidant. Even in this case, the AOC of the extract (1042.0 mg/L: 59.13 ± 0.3 mM and 38.6 mg/L: 4.69 ± 0.1 mM TEAC) was greater than that of the NPs (1042.0 mg/L NPs: 32.2 ± 0.4 mM and 38.6 mg/L NPs: 2.31 ± 0.01 mM TEAC); however, empty NPs showed negligible reducing power (0.06 ± 0.01 mM TEAC). pH is a critical point in such colourimetric assays. In both cases, the pH of the working solution was adjusted to pH 4.0 to avoid any modification of the NP structure, especially in light of the results shown in Figure 7. This pH value ensures both the solubility equilibrium of the Fe^3+^/Fe^2+^ species, which precipitate at neutral conditions, and the stability of the ABTS^•+^ radical, which is less prone to spontaneous auto-degradation [35].

### 3.3. Fourier-Transform IR (FT-IR) Analysis of Coacervates

**Figure 9:**
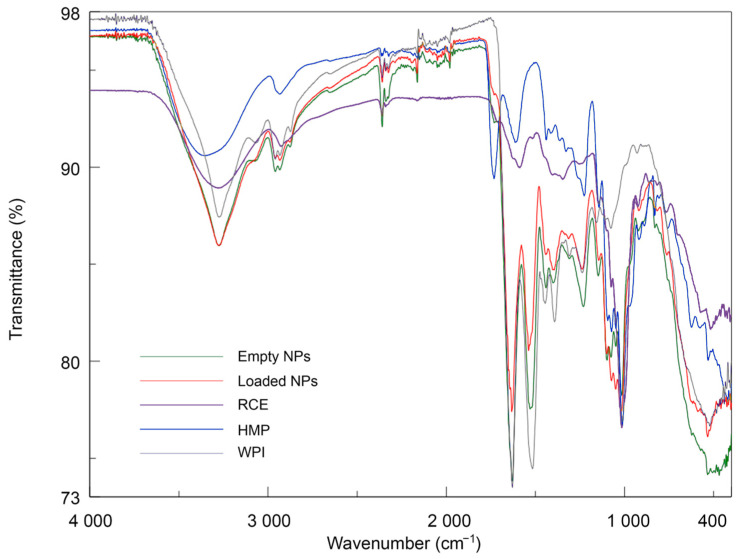
IR spectra of WPI, HMP, RCE, empty NPs, and RCE-loaded NPs.

Infrared spectroscopy is an analytical technique that provides indications of the presence of specific functional groups. In this study, there is evidence of particular structural moieties, including peptide bonds in WPI, polysaccharides composed of galacturonic acid partially converted into methyl ester in HMP, and glycosylated ACNs present in RCE, highly acylated by hydroxycinnamoyl residues such as feruloyl, *p*-coumaroyl, and sinapoyl, as determined by HPLC-DAD and MS analysis (Figure 1 and Table 1). The IR spectra registered within the 400–4000 cm^−1^ range of WPI, HMP, RCE, empty NPs, and RCE-loaded NPs are presented in Figure 9.

WPI displayed the typical protein spectrum, showing a large band between 3600 and 3000 cm^−1^ assigned to O-H bonds stretching with a minimum at 3276 cm^−1^ due to N-H stretching (Amide A band) and the two main bands at 1631 cm^−1^ arising mainly from the C=O stretching vibration (Amide I band) and at 1515 cm^−1^ due to in-plane N-H bending and C-N stretching vibration (Amide II band) [36]. Minor peaks below 3000 cm^−1^ (2960 and 2930 cm^−1^) are relative to C-H stretching. The HMP spectrum was characterised by a broad absorption band at around 3358 cm^−1^ due to OH stretching with a remarkable intensity, which is typical of polyhydroxyl compounds; a band of low intensity at around 2900 cm^−1^ due to C-H stretching, and two characteristic peaks at 1730 cm^−1^ and 1612 assigned to the C=O stretchings of methoxyl groups and carboxyl groups, respectively [37,38]. The peak at 1010 cm^−1^ is probably attributable to the glycosidic linkage between sugar units.

The RCE spectrum showed typical absorption for anthocyanins, characterised by the broad O-H stretching of the phenyl units at 3276 cm^−1^, the very weak C-H stretching at 2930 cm^−1^, the weak band at 1587 probably due to C=C stretching vibration of the aromatic rings, typically absorbing around 1600 cm^−1^ [39], and the intense absorption at around 530 cm^−1^ in the fingerprint region. Additionally, the intense peak at 1013 cm^−1^, attributable to C-O stretching of the glycosidic units, and the broadband at 3276 cm^−1^, are indicative of glycosyl-substituted anthocyanins.

Empty NPs showed an IR spectrum markedly more similar to that of WPI than to methylated pectin. Indeed, while the two Amide bands are well visible, the C=O vibration of the methyl ester group produced a faint signal. The ratio of WPI:HMP is 5:2, not high enough to justify such a difference in the band intensities. In addition, the frequencies of the O-H and Amide I bands remained almost unaltered, and only the Amide II band showed a shift of 9 cm^−1^ (1525 cm^−1^). Similar results were observed by Guerrero et al. (2014) [40], who studied the effects of blending different carboxylated polysaccharides with model proteins. When polysaccharides were added, the vibrational frequencies assigned to the amide I and II bands remained constant, while the band assigned to carboxylate in the polysaccharides was not detectable in the blends with proteins. This indicates the occurrence of interactions between the protein and the polysaccharides, highlighting the lower capacity of the carbohydrates to form intermolecular hydrogen bonds between themselves in the presence of the protein [41]. The spectrum of RCE-loaded NPs qualitatively resembled that of the empty ones: the O-H stretching band is characterised by the same frequency but with a higher intensity. Moreover, the band assigned to C-OH at 1013 cm^−1^ showed a markedly higher intensity with respect to empty NPs. The rest of the profile, in particular the Amide I and Amide II bands’ absorption, is less intense, and the contribution of C=C-C vibration is not appreciable. The absorption at 2960 cm^−1^ is less intense; this peak is specific to the polymers, and the addition of RCE reduced its contribution to the spectrum. The results indicate that between WPI, HMP, and the phenolic compounds contained in the extract, there are some interactions.

### 3.4. XRPD Analysis

**Figure 10:**
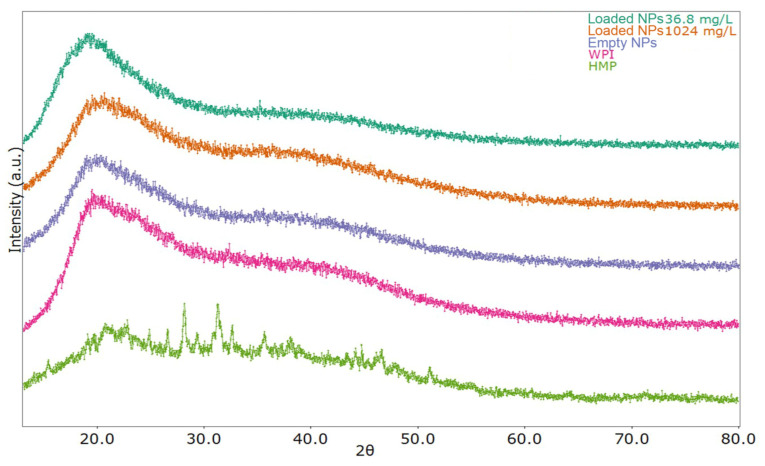
XRPD spectra of WPI, HMP, empty NPs, and RCE-loaded NPs.

The result of X-ray diffraction is presented in Figure 10. All the powder patterns (with the exception of data collected on pure HMP) exhibit typical features of amorphous substances, with a broad, diffuse signal centred at about 20° and another extended shoulder located around 40°, clearly indicating the absence of a three-dimensional long-range order in the sample volume. Similar results have been previously described for WPI-based nanoparticles [42]. Minor variations in the position, intensity, and shape of the diffuse signals can be ascribed mainly to sample morphologies due to different powder compressibility. On the other hand, as already observed [43], the diffraction pattern of the pure HMP sample clearly shows, in addition to the diffuse background, several sharp peaks due to Bragg scattering, indicating the presence of one or more crystalline or nanocrystalline substances; in particular, the strongest diffraction lines are located at 25°, 26.5°, 28°, 29.5°, 31.3°, and 32.5°. The absence of any visible Bragg signals from the diffraction patterns of the other HMP-containing compounds suggests that such weight percentages are below the detectability limit for the technique, likely due to an average crystallite size decrease after the nanoencapsulation process.

### 3.5. Encapsulation Efficiency

The encapsulation efficiency (EE%) was assessed through HPLC-DAD after the removal of unbound molecules and the denaturation of the coacervates with the release of encapsulated ACNs. Due to the presence of several different ACNs in RCE, the EE% was calculated as the sum of all quantified peaks normalised by their relative abundance. The EE% was found to be 29.3% ± 0.1, in accordance with the results presented by Arroyo-Maya and McClements [11], which attested their loading efficiency between 17% ± 5 and 35% ± 7 for the NPs prepared by adding the ACNs extract after the thermal treatment. The comparison of the HPLC profiles of the NP content before and after dialysis revealed peculiar binding profiles of the ACNs to the polymeric network in agreement with the FTIR results.

**Figure 11:**
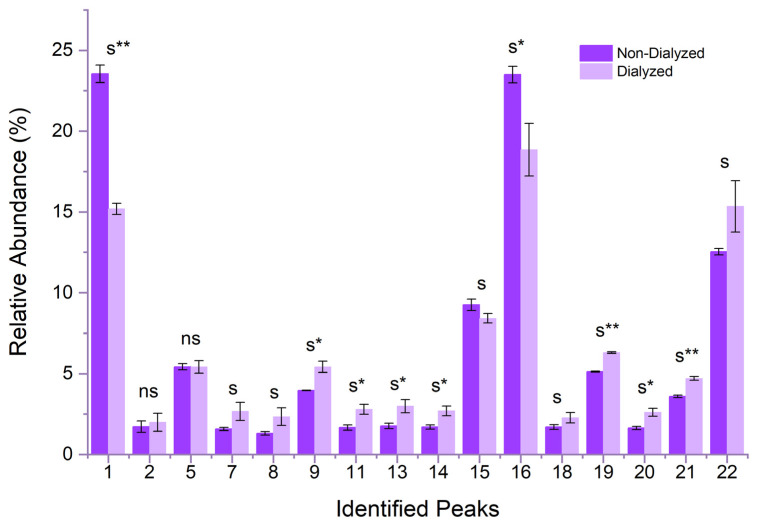
Relative abundance of individual ACNs loaded in the NPs before and after dialysis. ACNs are indicated with the same numbering as in Figure 1. The significance attribution was given based on the results of an unequal variances *t*-test (Welch *t*-test) performed on each dialysed and non-dialysed peak, normalised over the respective total ACNs content, with S* and S** indicating significant differences with *p* values ≤ 10^−3^ and ≤10^−6^, respectively. ns indicates non-significant differences.

A comparison of the ratio between each single ACN and the total amount of all ACNs contained in the extract showed that during dialysis, the molecules harbouring a triglycosylation on the C_3_ position and/or a double acylation are retained more efficiently within the biopolymer than the ACNs presenting a disaccharide or a monoacylation (Figure 11). In almost all cases, the relative concentration of all peaks differed significantly from their respective non-dialysed homologues, except for peaks Nos. 2 and 5, whose concentrations did not show any enrichment or depletion. For peak No. 5, this could be due to the coelution of two ACNs, cyanidin-3-(sinapoyl)-diglucoside-5-glucoside and cyanidin-3-(sinapoyl)-triglucoside-5-glucoside. Notably, the non-acylated form, cyanidin-3-diglucoside-5-glucoside, was reduced by nearly 40% after dialysis, highlighting the weak interaction established with the polymeric structure. A complex interplay of different factors, including the degree of glycosylation and/or acylation, appears to affect the affinity of each ACN to the polymers of the coacervates and, in turn, the overall encapsulation efficiency and stability of the molecules. Indeed, as we recently described for silver linden (*Tilia tomentosa*) flavonoids, the stability of encapsulated molecules is positively influenced by the interaction with the matrix [44]. To the best of our knowledge, this is the first time that the loading of a coacervate with a complex phenolic mixture is characterised at the single-molecule level. Further investigations are required to elucidate the mechanism underlying the differential retention of each ACN within the coacervate polymeric structure.

## 4. Conclusions

The present study described a simple and sustainable coacervation process to nanoencapsulate RC ACNs starting from two by-products of the food industry, i.e., WPI and pectin, thus contributing to their valorisation. The pH adjustment to 4.0 led to the assembly of a monodispersed population of NPs whose dimension and ζ-potential are influenced by the concentration of the extract. Using an HPLC approach, we could provide insight into the binding affinity between the biopolymeric network and the different ACNs, showing a major binding capacity of the more glycosylated and acylated ACNs over the others. The influence of structure on the interaction with the network might be based on the affinity between the acyl groups/sugar moieties and the polymers or might be stereochemically driven by their more extended configuration. Further studies on the binding of single ACNs from different plant sources with the polymeric network could help verify whether their structure affects the encapsulation efficiency and predict which anthocyanins are better suited to be vehiculated by this approach.

## Figures and Tables

**Table 2:** Z-Average, PDI (polydispersity index), diameter, and ζ-potential of the nanoparticles developed with the different methods.

Method	Blending Order	Heat Treatment	Z-Average (nm)	PDI	Diameter (nm)	ζ-Potential (mV)
1	WPI + RCE + HMP (1:0.2:1)	None	396.80 ± 21.78	0.225 ± 0.03	385.85 ± 7.26 a	−16.83 ± 0.33 ab
2	WPI + RCE + HMP (1:0.2:1)	WPI, 65 °C, before blending	323.71 ± 12.97	0.170 ± 0.09	337.28 ± 5.33 b	−17.76 ± 0.40 a
3	WPI + HMP + RCE (1:1:0.2)	WPI, 65 °C before blending	428.61 ± 35.35	0.308 ± 0.04	384.31 ± 11.69 a	−17.99 ± 0.15 a
4	WPI + HMP + RCE (1:1:0.2)	Whole mix, 65 °C, after blending	507.62 ± 13.8	0.283 ± 0.03	445.20 ± 2.23 c	−15.86 ± 0.38 b

Data are expressed as the mean ± standard error (SE) from triplicates. Different lowercase letters within the same column indicate significant differences (*p* ≤ 0.05). The relative volume ratio of WPI, RCE, and HMP used for every formulation is reported in round brackets. The ACN concentration was 38.6 mg/L. Data for methods 5 and 6, which required the application of a 90 °C thermal treatment, are not shown due to the coagulation of the WPI solution during the coacervation process.

**Table 3:** Z-Average, PDI (polydispersity index), the diameter of the main peak, and ζ-potential of the nanoparticles developed with different concentrations of RCE using method No. 2.

ACN Concentration (mg/L)	Z-Average (nm)	PDI	Diameter (nm)	ζ-Potential (mV)
0	320.4 ± 5.2	0.09 ± 0.1	337.2 ± 6.7 a	−17.20 ± 1.0 a
38.6	323.7 ± 13.0	0.170 ± 0.1	337.3 ± 5.3 a	−15.64 ± 0.0 b
115.9	322.7 ± 9.7	0.160 ± 0.1	335.9 ± 6.4 a	−15.52 ± 0.1 b
347.4	343.0 ± 2.1	0.050 ± 0.0	356.8 ± 0.4 b	−15.34 ± 0.1 b
1042.0	365.0 ± 8.5	0.250 ± 0.1	368.2 ± 3.0 b	−14.24 ± 0.3 c

Data are expressed as the mean ± standard error (SE) from triplicates. Different lowercase letters within the same column indicate significant differences (*p* ≤ 0.05).

## Data Availability

Data are available on request from the authors.
